# Prognostic impact of cardiac resynchronization therapy guided by phase analysis: a CZT study

**DOI:** 10.1093/ehjimp/qyad004

**Published:** 2023-06-19

**Authors:** Riccardo Liga, Umberto Startari, Davide Spatafora, Erica Michelotti, Alessia Gimelli

**Affiliations:** Dipartimento di Patologia Chirurgica, Medica, Molecolare e dell’Area Critica, University of Pisa, Pisa, Italy; Cardio-thoracic and Vascular Department, University Hospital of Pisa, Pisa, Italy; Fondazione Toscana Gabriele Monasterio, Via Moruzzi, 1, 56124 Pisa, Italy; Cardio-thoracic and Vascular Department, University Hospital of Pisa, Pisa, Italy; Cardio-thoracic and Vascular Department, University Hospital of Pisa, Pisa, Italy; Fondazione Toscana Gabriele Monasterio, Via Moruzzi, 1, 56124 Pisa, Italy

**Keywords:** mechanical dyssynchrony, phase analysis, CZT, cardiac resynchronization therapy, myocardial perfusion imaging, pacing

## Abstract

**Aims:**

To evaluate whether phase analysis imaging may predict treatment response and long-term prognosis after cardiac resynchronization therapy (CRT).

**Methods and results:**

Sixty-nine patients underwent myocardial perfusion imaging followed by CRT. Patients with ischaemic heart disease and non-ischaemic cardiomyopathy (NICM) were identified. Left ventricular (LV) mechanical dyssynchrony (LVMD) was assessed at phase analysis and the region of the latest mechanical activation was identified. LV pacing lead position was considered ‘concordant’ when located in the region of the latest mechanical activation, and ‘discordant’ otherwise. The ‘6 months post-CRT’/‘baseline’ ratio of LV ejection fraction was computed as a measure of CRT response. LVMD was revealed in 47/69 patients, 27 of whom (57%) had a concordant LV lead implantation. Only concordant pacing was associated with LV functional improvement (ejection fraction ratio: 1.28 ± 0.25 vs. 1.11 ± 0.32 in discordant stimulation, *P* = 0.028). However, this relationship persisted only in patients with NICM (*P* < 0.001), while it disappeared in those with ischaemic heart disease (*P* = NS). Twenty-eight events occurred during 30 ± 21 months follow-up. While discordant LV lead location was the major predictor of unfavourable prognosis (hazard ratio 3.29, 95% confidence interval 1.25–8.72; *P* = 0.016), this relationship was confirmed only in patients with NICM.

**Conclusions:**

Phase analysis of myocardial perfusion imaging may guide CRT implantation, identifying patients who would most likely benefit from this procedure.

## Graphical Abstract

**Graphical abstract:**
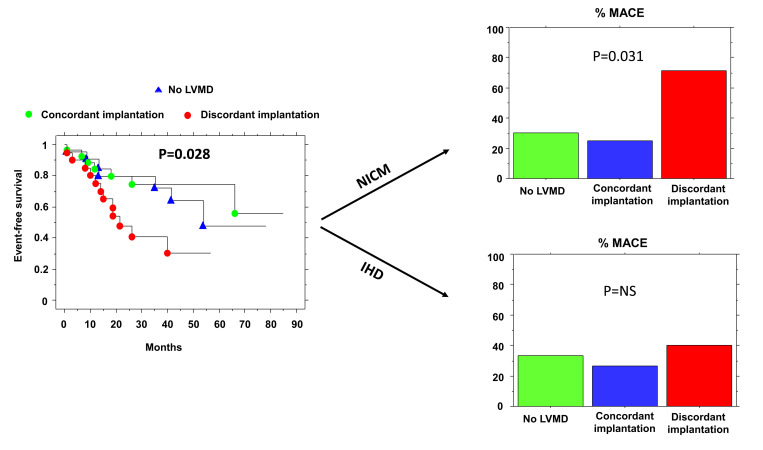
Impact of the presence of LVMD and of LV stimulation patterns (i.e. concordant and discordant LV lead placement) on long-term prognosis in the overall population (left) and according to heart failure aetiology (right). IHD, ischaemic heart disease; LVMD, left ventricular mechanical dyssynchrony; NICM, non-ischaemic cardiomyopathy.

## Introduction

Cardiac resynchronization therapy (CRT) is an effective strategy for the treatment of patients with heart failure (HF) and reduced (r) ejection fraction (EF).^1^ Moreover, CRT is also indicated in patients with midrange (mr) EF (between 40 and 50%) who have an indication of anti-bradycardia pacing.^2^ However, a consistent proportion of patients are ‘non-responders’ to treatment.^3^ Phase analysis (PA) on single-photon emission computed tomography myocardial perfusion imaging (MPI) allows the absolute quantification of left ventricular (LV) mechanical dyssynchrony (MD).^4^ Moreover, given the combined evaluation of regional LV perfusion, PA on single-photon emission computed tomography MPI may individuate the ideal zone for LV lead placement.^5,6^

However, no information is available regarding the long-term prognostic impact of PA parameters in patients submitted to CRT. We aimed to evaluate the value of PA-derived LVMD assessment in predicting treatment response and long-term prognosis in patients submitted to CRT device implantation.

## Methods

### Patient population

Sixty-nine consecutive subjects were submitted to MPI on a Cadmium–Zinc–Telluride (CZT) camera 1–3 months before the procedure between 2012 and 2020. Patients with ischaemic heart disease (IHD) were distinguished from those with non-ischaemic cardiomyopathy (NICM).^7^ Exclusion criteria were haemodynamic instability, myocardial inflammatory disease, and moderate valvular disease. The study was approved by the Local Ethical Committee and conformed to the Declaration of Helsinki on human research. Written informed consent was obtained from every patient after an explanation of the protocol, its aims, and potential risks.

### Acquisition protocol

Patients underwent CZT (Discovery NM 530c; GE Healthcare, Haifa, Israel) imaging according to a single-day protocol, as previously reported.^4,8^ Images were reconstructed without scatter or attenuation correction. Perfusion images were scored according to the 17-segment LV model and a five-point scale (0 = normal, 1 = equivocal, 2 = moderate, 3 = severe reduction in radioisotope uptake, and 4 = absence of detectable tracer uptake), with a grade ≥2 indicating a scar.

### Analysis of gated images

PA was performed on 16-frames reformatted CZT images using commercially available software (Corridor4DM, Invia, Ann Arbor, MI, USA). The standard deviation (SD) of the phase distribution and the histogram bandwidth were automatically generated as measures of LVMD.^9^ The presence of LVMD was diagnosed when the phase bandwidth and/or the SD exceeded by two SDs, the previously published reference values for healthy men (bandwidth 89° ± 38°; SD 20° ± 10°) and women (bandwidth 88° ± 31°; SD 21° ± 11°) obtained with the same CZT device and imaging protocol.^4^ In patients with LVMD, the region of the latest mechanical activation of the LV myocardium was automatically identified. When the latest contracting region was represented by the apex, the most delayed nearby myocardial wall was considered for subsequent analyses.

### Clinical evaluation and echocardiography

Patients were clinically evaluated at baseline and 6 months after CRT implantation. HF symptoms were classified using the New York Heart Association (NYHA) classification. A surface 12-lead electrocardiogram (ECG) was obtained at all visits and QRS duration was measured. Left-bundle branch block was defined in agreement with the criteria proposed by Strauss *et al*.^10^ A two-dimensional echocardiogram (2D-Echo) was performed at baseline and repeated 6 months after CRT device implantation. The LV end-diastolic (EDV) and end-systolic volumes were calculated, and the LVEF was derived accordingly. In each patient, the 6 months post-CRT/baseline ratios of QRS duration, LVEF, and LV EDV index were computed as measures of CRT response. The occurrence of a favourable response to CRT stimulation was recognized in patients showing a ≥ 5% increase in LVEF and/or ≥10% reduction in the EDV.

### Determination of LV lead position

After implantation, the LV lead position was assessed from a chest X-ray, as described previously.^6^ LV-pacing lead position was considered concordant when located on the region of latest mechanical activation, and discordant otherwise.

### Follow-up

Patients were followed with periodic clinical evaluations and/or telephone interviews every 6 months after CRT implantation. The occurrence of major adverse cardiac events (MACE)—comprising cardiac death, acute coronary syndrome, and urgent hospitalization for cardiac causes—was recorded.

### Statistical analysis

Continuous variables were expressed as mean ± SD and categorical variables as percentages. All tests were two-sided; a *P* < 0.05 was considered significant. The predictors of MACE were assessed at univariable and multivariable COX regression analysis and the hazard ratios (HRs) with the relative 95% confidence intervals (CIs) were determined. Statistical analyses were performed using JMP (SAS Institute Inc, version 4.0.0) and Stata (version 13, StataCorp, College Station, TX, USA).

## Results

At CZT evaluation, 47 patients had significant LVMD while 22 patients had not. In patients with LVMD, the LV region showing the latest mechanical activation was located in the anterior, antero-lateral, infero-lateral, and inferior in 7, 30, 8, and 2 patients, respectively, and was scarred in 22 of them (82% with IHD). On the other hand, the pacing LV lead was located at the level of the anterior, antero-lateral, infero-lateral, and inferior walls in 2, 15, 26, and 4 patients, respectively. In 27/47 LVMD patients (57%), the LV lead was implanted in the region with the latest mechanical activation (concordant), while in the remaining 20/47 patients (43%), it was not (discordant).

### CRT on clinical and LV functional parameters

After 6 months from implantation 36/69 (52%) of patients were responders to CRT stimulation, while the remaining 33/69 (48%) patients were non-responders. No differences in major demographic, clinical, and ECG criteria were observed between the two groups of patients (*Table 1*). Conversely, responders to CRT had a significantly lower LVEF and higher prevalence of concordant LV lead placement than non-responders (*Table 1*).

**Table 1:** Clinical characteristics

Parameters	Study patients (*n* = 69)	CRT non-responders (*n* = 33)	CRT responders (*n* = 36)	*P*-value
Age, years	81 ± 8	81 ± 8	80 ± 9	0.695
Males, *n* (%)	59 (86)	29 (88)	30 (83)	0.737
Previous myocardial infarction, *n* (%)	32 (46)	14 (42)	18 (50)	0.777
NYHA class	2.0 ± 1.0	2.0 ± 1.0	2.5 ± 1.0	0.531
Indications for CRT implantation, *n* (%)				0.088
HFrEF refractory to OMT	43 (62)	17 (52)	26 (72)	
HFmrEF undergoing anti-bradycardia pacing	26 (38)	16 (48)	10 (28)	
ECG parameters				
Atrial fibrillation, *n* (%)	46 (67)	23 (70)	23 (59)	0.798
QRS duration, ms	134 ± 23	133 ± 24	135 ± 23	0.761
QRS morphology, *n* (%)				0.999
Left-bundle branch block	17 (25)	8 (24)	9 (25)	
Right bundle branch block	7 (10)	3 (9)	4 (11)	
Intraventricular conduction delay	19 (28)	6 (18)	13 (36)	
Echo data				
End-diastolic volume index, mL/m^2^	82 ± 25	81 ± 23	83 ± 27	0.673
Ejection fraction, %	37 ± 8	39 ± 8	35 ± 8	0.015
Mitral regurgitation (≥moderate), *n* (%)	7 (10)	3 (9)	4 (11)	0.999
Dyssynchrony analysis				0.011
No LVMD, *n* (%)	22 (32)	13 (39)	9 (25)	
Discordant stimulation, *n* (%)	20 (29)	13 (39)	7 (19)	
Concordant stimulation, *n* (%)	27 (49)	7 (22)	20 (56)	
Pharmacological therapy				
Beta-blockers, *n* (%)	61 (88)	33 (100)	36 (100)	
RAS inhibitor, %	50 (62)	23 (70)	27 (75)	
Mineralcorticoid antagonist, *n* (%)	27 (39)	13 (39)	14 (39)	
Diuretics, *n* (%)	58 (84)	28 (85)	30 (83)	
SGLT2 inhibitors, *n* (%)	12 (17)	5 (15)	7 (19)	

HFrEF: heart failure with reduced ejection fraction; HFmrEF: heart failure with mid-range ejection fraction.

As shown in Supplementary data online, *Table S1*, seven patients with discordant LV lead placement responded to CRT stimulation, three of whom had a history of MI (lateral in two and inferior one of them).

### Patterns of CRT stimulation and LV functional parameters

**Figure 1:**
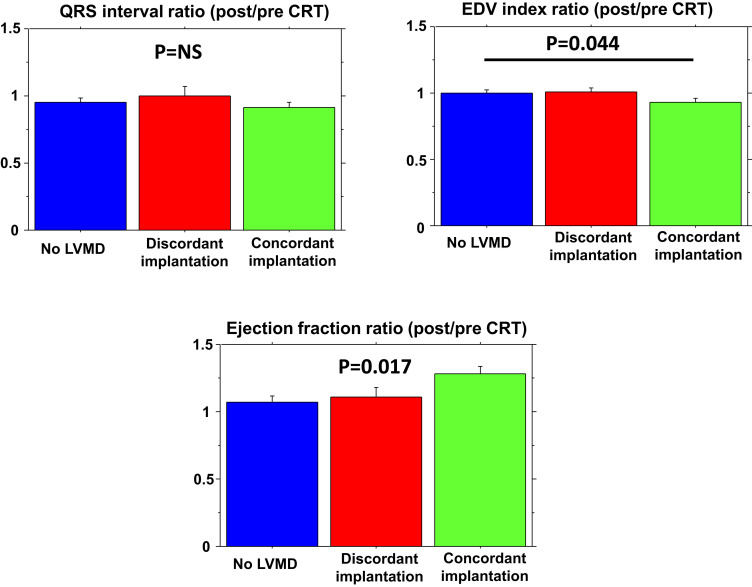
Impact of CRT on major cardiac functional parameters according to the presence of LVMD and LV stimulation patterns (i.e. concordant and discordant LV lead placement).

The impact of CRT stimulation on LV structure and function at follow-up is reported in *Figure 1*. Only concordant CRT stimulation was associated with LV functional improvement when compared with baseline. However, when the association between HF aetiology and CRT response was evaluated, only patients with NICM and concordant LV stimulation showed consistent LV reverse remodelling, while no such effect was demonstrated in those with IHD (*Table 2*). The significant association between concordant LV stimulation and favourable CRT response was confirmed also in patients without Left Bundle Branch Block (LBBB) morphology at ECG (responders 72 vs. 33% in discordant LV stimulation, *P* = 0.045).

**Table 2:** Interaction between LV lead location, HF aetiology, and response to CRT

Parameters	IHD					NICM		
	No LVMD (*n* = 12)	Discordant LV lead (*n* = 5)	Concordant LV lead (*n* = 15)	*P*-value	No LVMD (*n* = 10)	Discordant LV lead (*n* = 15)	Concordant LV lead (*n* = 12)	*P*-value
Δ NYHA class	0.5 ± 1	1 ± 0	0 ± 1	0.301	0 ± 0	0 ± 1	1 ± 2	0.031
QRS duration ratio	0.88 ± 0.08	0.99 ± 0.41	0.99 ± 0.20	0.432	1.03 ± 0.15	1.00 ± 0.27	0.82 ± 0.12	0.033
LVEF ratio	1.10 ± 0.20	1.45 ± 0.37	1.17 ± 0.21	0.031	1.04 ± 0.22	0.98 ± 0.20	1.43 ± 0.30	<0.001
EDVind ratio	1.03 ± 0.11	0.94 ± 0.20	0.98 ± 0.14	0.465	0.96 ± 0.12	1.04 ± 0.13	0.85 ± 0.15	0.010

Δ(delta): difference between the pre-CRT and post-CRT value; ratio: ‘post/pre’-CRT value.

### Follow-up analysis

The mean follow-up was 30 ± 21 months. Twenty-eight MACEs were recorded in 26 patients, including 4 cardiac deaths, and 24 urgent hospitalizations for cardiac causes. At COX analysis, including major clinical and LV functional variables, a discordant LV lead location was the major predictor of unfavourable prognosis at follow-up (HR 3.29, 95% CI 1.25–8.72; *P* = 0.016). However, this association was confirmed only in patients with NICM, while disappeared in those with IHD (*Graphical abstract*).

## Discussion

The present study shows that PA performed on a CZT cardiac camera may guide CRT implantation, predicting patients that would most likely benefit from this procedure. In NICM patients, in whom LV lead was concordantly placed at the level of the most dyssynchronous myocardial region, reverse LV remodelling can be expected, associated with significantly improved long-term outcomes. Conversely, patients with IHD do not seem to benefit from CRT implantation, showing an overall poor functional and clinical response to treatment, likely because of the unfavourable impact of myocardial scar.

Our results seem of particular interest considering that recent appraisals have confirmed how a consistent proportion of HF patients does not benefit from CRT,^3^ raising doubts on the validity of current criteria for device implantation. Present data show that when the LV lead is implanted in the viable region with the latest mechanical activation a favourable prognosis can be expected, even at prolonged follow-up. In a subset of patients with discordant LV lead placement (43% of whom had suffered a previous MI), a favourable response to CRT was observed, likely because of the intentional stimulation of a ‘less dyssynchronous’ but viable myocardial region.

In our study, this favourable trend remained true even after including patients with less severe LV functional impairment (EF >35%) that represent an increasing population of subjects possibly candidates for CRT.^2^ In this regard, while responders to CRT stimulation had a modestly lower LVEF at baseline, on multivariate analysis the presence of a concordant stimulation (odds ratio 7.67, 95% CI 1.41–41.63, *P* = 0.018) remained an independent predictor of a favourable LV functional response, overwhelming other clinical, ECG, and cardiac functional parameters.

The relatively heterogeneous population and limited study numbers may prevent any solid causal inference among the variables explored. However, the characteristics of our patients closely mirror those of the subjects that are currently evaluated for CRT in clinical practice.

## Conclusions

When CRT implantation is considered, MPI may offer an integrated evaluation of LVMD and regional LV perfusion, localizing the viable myocardial region with the latest mechanical activation and individuating those patients in whom functional and prognostic benefits can be expected.

## Supplementary Material

qyad004_Supplementary_Data

## Data Availability

The data underlying this article will be shared on reasonable request to the corresponding author.

## Lead author biography

Dr Riccardo Liga is a cardiologist working at the University Hospital of Pisa and a Researcher of the University of Pisa. He is mostly interested in multimodality cardiac imaging, with a special focus on nuclear cardiology techniques and cardiac CT. He currently serves as board member of the ‘European Association of Cardiovascular Imaging’ and is an Editorial Board member of the *European Heart Journal—Case Reports* and *European Heart Journal—Imaging Methods and Practice* Journals.
