# Supplementary material for: Prognostic impact of cardiac resynchronization therapy guided by phase analysis: a CZT study
Source: Eur Heart J Imaging Methods Pract. 2023 Jun 19;1(1):qyad004. doi: 10.1093/ehjimp/qyad004 (PMC11195782; doi:10.1093/ehjimp/qyad004)
Supplement: qyad004_Supplementary_Data [file qyad004_Supplementary_Data.doc]

**Suppl. Table 1. Characteristics of responders to CRT with “discordant” stimulation**

| **Parameters** | **“Discordant” stimulation (N=7)** |
| --- | --- |
| Age, (yrs) | 79±11 |
| Males, n | 7 |
| Previous myocardial infarction, n | 3 |
| Indications for CRT implantation, n |  |
| - *HFrEF refractory to OMT* | 5 |
| - *HFmrEF undergoing anti-bradycardia pacing* | 2 |
| ***ECG parameters*** |  |
| Atrial fibrillation, n | 6 |
| QRS duration, msec | 123±28 |
| QRS morphology, n (%) |  |
| - *Left bundle branch block* | 1 |
| - *Right bundle branch block* | 1 |
| - *Intraventricular conduction delay* | 5 |
| **Echo parameters** | |
| End-diastolic volume index, ml/m^2^ | 79±16 |
| Ejection fraction, % | 34±9 |
| **Dyssynchronous LV wall** |  |
| Inferior, n | 2 |
| Infero-lateral, n | 2 |
| Lateral, n | 3 |
| Anterior, n | 0 |
| ***LV lead location*** |  |
| Inferior, n | 1 |
| Infero-lateral, n | 2 |
| Lateral, n | 2 |
| Anterior, n | 2 |
